# The future of value in digitalised higher education: why data privacy should not be our biggest concern

**DOI:** 10.1007/s10734-020-00639-7

**Published:** 2020-11-19

**Authors:** Janja Komljenovic

**Affiliations:** Educational Research, Lancaster University, Lancaster, UK

**Keywords:** Higher education industry, Value, Assetization, Marketization, Rent

## Abstract

Universities around the world are increasingly digitalising all of their operations, with the current COVID-19 pandemic speeding up otherwise steady developments. This article focuses on the political economy of higher education (HE) digitalisation and suggests a new research programme. I foreground three principal arguments, which are empirically, theoretically, and politically crucial for HE scholars. First, most literature is examining the impacts of digitalisation on the HE sector and its subjects alone. I argue that current changes in digitalising HE cannot be studied in isolation from broader changes in the global economy. Specifically, HE digitalisation is embedded in the expansion of the digital economy, which is marked by new forms of value extraction and rentiership. Second, the emerging research on the intersection of marketisation and digitalisation in HE seems to follow the theories of marketisation qua production and commodification. I argue that we need theories with better explanatory power in analysing the current digitalisation dynamics. I propose to move from commodification to assetisation, and from prices to rents. Finally, universities are digitalising in the time when the practice is superseding policy, and there is no regulation beyond the question of data privacy. However, digital data property is already a reality, governed by ‘terms of use’, and protected by the intellectual property rights regime. The current pandemic has led to ‘emergency pedagogy’, which has intensified overall digitalisation in the sector and is bypassing concerns of data value redistribution. I argue that we urgently need public scrutiny and political action to address issues of value extraction and redistribution in HE.

## Introduction

Universities around the world are increasingly digitalising all of their operations. Delivering teaching and conducting research online, making decisions based on learning and business analytics, and turning campuses ‘smart’ are only a few examples of an incredibly vibrant and expanding ecosystem of digital platforms in higher education (HE). Universities do not digitalise alone and use proprietary digital platforms for their everyday operations. They also partner with companies, such as providers of Online Programme Management (OPM) and Massive Open Online Course (MOOC) platforms to deliver full or parts of study programmes online. Moreover, new digital products and services are targeting students and staff directly, which are complementing university functions or challenging them entirely. Digital technological innovations might improve HE and bring benefits to students, academics, and university administrators. However, they also bring new monetisation opportunities, which is the focus of this article. There is much research on technology-enhanced learning, which is mainly investigating the impact of certain technologies on a specific aspect of teaching and learning processes. But there is far less research on monetisation, privatisation, and profit concerning HE digitalisation. Indeed, the numbers on the education technology (EdTech) industry’s worth and growth are impressive and deserving of our attention.^1^

EdTech companies have become key actors in HE with the current COVID-19 pandemic speeding up otherwise steady developments in HE digitalisation by 5 to 10 years.^2^ While investment in EdTech has been rising to unprecedented amounts already before the pandemic, the investors’ interest in education has been intensified since. Moreover, the pandemic has removed potential resistance to HE digitalisation and allowed for what was called the world’s greatest experiment for online teaching and learning (Williamson & Hogan, 2020). It is said that HE will never be the same after the pandemic, with most commentators predicting a form of blended teaching and learning to stay. We are witnessing new ways and types of privatisation and marketisation in HE through digitalisation. Therefore, we cannot separate the digitalisation and marketisation of universities (Williamson, 2018).

The study of HE markets has so far almost entirely focused on service-commodities (for example, Brown, 2011; Jungblut & Vukasovic, 2018; Marginson, 2013, 2018; Naidoo & Williams, 2015). This is including emerging studies on digitalisation of HE (Castañeda & Selwyn, 2018; Perrotta, 2018; Swartz, Ivancheva, Czerniewicz, & Morris, 2019; Swinnerton et al., 2019; Williamson, 2018). However, digitalisation of HE involves production and extraction of digital data, and data and data products do not act like commodities. Commodities are consumed once used, but digital data is reproducible at almost zero marginal cost (Savona, 2019). New products and services can be created from digital data and monetised through subscription fees, an app, or a platform that does not transfer ownership, control, or reproduction rights to the user (Birch, 2020). Furthermore, data use creates yet more data, and the network effects increase the value of these platforms (Srnicek, 2017). Therefore, there is a new quality at play in the monetisation and marketisation of these digital HE products and services, which is different from the commodity form. We are witnessing a widespread change from creating value via market exchange towards extracting value via the ownership and control of assets (Mazzucato, 2018).

This article is conceptual. It proposes a new theoretical framework to analyse HE digitalisation and marketisation and suggests a future research agenda for HE scholars. In what follows, I first present the emerging literature on the intersection of digitalisation and marketisation of HE. I then discuss the empirical, theoretical, and political moves, which I argue are necessary for the future research of HE digitalisation. In the empirical move, I present the shifts brought by the digital economy and their impacts on the HE sector. I suggest that our research focus should turn to the variety of digitalisation forms in HE involving different actors and monetisation models. In the theoretical move, I distinguish between commodities and assets. I present Birch’s theory of rentiership as a useful framework to study data rents in HE (Birch, 2020). In the political move, I discuss why our attention should not be only on data privacy but also on the data value. HE policy-makers urgently need research to underpin their decision-making on HE digitalisation and its regulation.

### On the intersection of digitalisation and marketisation

The research on the intersection of marketisation and digitalisation of HE is novel, and the literature is only emerging. The first line of the developing literature on these processes examines the increasing use of digital data for governing and marketising the HE sector. These studies are mainly focused on the UK. The state-organised capturing of the student data is used to measure, compare, and assess university performance (Holmwood & Marcuello Servós, 2019; Williamson, 2019). While digital technology is advancing neoliberal forms of metric power in HE (Williamson, Bayne, & Shay, 2020), HE digital infrastructure acts as a hidden architecture for the marketisation of the sector (Williamson, 2018). Political aims and business objectives are becoming aligned as common aspirations (Williamson, 2019). Not only is the technology used for market-making in the national HE system, but it is also envisaged that EdTech will strengthen British competitiveness in the global HE market (Munro, 2018). These studies reaffirm the role of the state in organising, supporting, and even funding marketisation initiatives in HE via digital technology.

The second group of emerging literature focuses on the presence of for-profit interests, or for-profit actors in digitalising HE, namely EdTech companies. The emerged global HE industry of digital data products uses data extracted from students for training machine learning systems of private companies (Williamson et al., 2020). Because these companies own the digital infrastructure and the digital data extracted by it, they became the sites of education research and creators of new education theories that are being built into the digital tools they sell to schools and universities (Williamson, 2017). Pearson, one of the biggest HE providers in the world, is introducing new types of learning based on demand-driven education, personalisation, and renting content, and creating new dependencies of public universities through its OPM (Williamson, 2020). Overall, digital technology is seen to advance neoliberal and free-market values in university practices, support monetisation of HE provision, and reconfigure it into a commodity state (Castañeda & Selwyn, 2018).

Finally, the impacts of digitalisation on HE are studied via partnerships between public universities, and MOOC and OPM platform companies (Thomas & Nedeva, 2018). These partnerships are found to reinforce university stratification and inequalities in the HE sectors that were analysed (Perrotta, 2018; Swinnerton et al., 2019). Moreover, unbundling of HE provision into its parts and reconfiguring it into new delivery forms is found to be one of the most significant impacts of digitalisation in HE (Cliff, Walji, Mogliacci, Morris, & Ivancheva, 2020; Swinnerton et al., 2019). University leaders find themselves in a conflict between generating new income streams and maintaining their social relevance as found by research in South Africa (Ivancheva et al., 2020).

These studies are incredibly rich and relevant and present only a start of the exploration into the complexities of HE markets and their digital dynamics. However, they stay in the realm of (i.) the economy as production instead of financialisation, (ii.) markets as commodification instead of assetisation, and (iii.) data concerns around privacy, but not value redistribution. These are the empirical, theoretical, and political points that I examine next.

### Empirical move: from the impact of digitalisation in higher education to platform imperative in the digital economy

HE is being digitalised in collaboration between universities and digital platform companies, albeit universities digitalise some of their operations alone. At the same time, companies also provide digital products and services directly to students and staff. The digitalisation of HE thus comes in very diverse forms (see Table 1). Collectively, digitalisation of HE is only part of the broader shift in the global economy towards the digital economy. In other words, the digital economy is not encompassing *only* but *also* the HE sector. HE researchers thus need to be attentive to the broader shifts in the economy while focusing on the specifics of HE within these shifts.

**Table 1:** Forms of digitalisation dynamics in the HE industry

Who	What	Examples	Monetisation model
Universities alone	Various digital products and services	Student and staff applications such as iLanaster developed by Lancaster University	No monetisation (internal cost for universities of in-house developers and infrastructure)
Universities and open source	Virtual learning environments, various digital products and services	Moodle	No monetisation (internal cost for universities of in-house developers and infrastructure)
Universities and companies (universities as users of platforms)	Various applications, platforms, e-resources (e.g. e-books), virtual learning environments, research management systems, various analytics and business intelligence software	Blackboard, Pure, Amazon Alexa for universities, learning analytics platforms, predictive learning platforms	Universities pay subscription fees, licences, or other fees (such as the number of clicks or time spent on platforms) to companies for access to the digital product or service, and for data intelligence
Universities and companies (partnerships)	Collaborative delivery of HE courses and modules	OPMs, MOOCs	Students pay fees for access and/or credentials; employers pay fees for recruitment or candidate screening; and other fees
Universities and state agencies	Various digital products and services	Jisc’s National Learning Analytics Service	Institutional subscriptions
State agencies and companies	Collaborative delivery of digital products and services	UCAS and Unibuddy, Jisc and start-up companies	Various combinations (institutional subscriptions, monthly access and visibility fees, state grants)
Companies alone (complementary to universities)	Developing products and services complementary to universities, and selling to students or other actors directly	Tutoring, online help with recruitment and admissions for students	Students pays fees or subscription
Companies alone (in competition with universities)	Developing products and services to challenge universities and compete	Alternative training and skills provision (for example coding), various micro-credentials	Various options and combinations—for example, a percentage of salary after getting employment, fees per course or credential, subscriptions
Companies together	Compatible and possibly collaborative products and services between platforms; integration via digital infrastructure	Commercial coalitions (for example, see Williamson & Hogan, 2020)	User pays fees for a service; or a subscription for continuous access

^a^Jisc is a UK not-for-profit membership organisation providing digital solutions for HE

^b^UCAS is the Universities and Colleges Admissions Service managing the application process for British universities. In 2019, it has partnered with an EdTech start-up company, Unibuddy, to connect student applicants with student ambassadors.

^c^Partnered with Emerge Education, Jisc developed a programme to support the university and EdTech start-up companies interaction including a ‘healthcheck’ of start-ups, and research and insights

"Note: The list is not exhaustive, but rather presents instantiations of digitalising the HE sector dynamics. There might be new categories that are under development, such as universities developing digital assets and offering them to other universities for subscription fees or similar charges. This is a matter of much needed empirical focus that I spell out in this and next section of the article."

#### Digital economy and value extraction

The digital economy is “that part of economic output derived solely or primarily from digital technologies with a business model based on digital goods or services” (Bukht & Heeks, 2017, p.13). In EdTech, this definition includes digital products and services such as Coursera, a MOOC platform, Aula, a learning experience platform for HE, or Amazon Alexa for universities. It differs from ‘digitisation’, which refers to “conversion of data from analogue to digital form” and ‘digitalisation’, which concerns the “application of digitisation to organisational and social processes (including economic activity)” (Bukht & Heeks, 2017, p.12). Most of the global economy is now digitalised, but not all is digital. Similarly, most of the HE sector is digitalised, but not all is digital. However, the business model based on digital products or services is fast expanding in HE.

The EdTech industry is a good proxy to grasp the extent of the digital economy’s presence in education. In early 2020, the global EdTech market was estimated at $186bn (IBIS Capital and Cairneagle Associates, 2020). Venture capital is increasingly interested in EdTech with steep growth of investment since 2016 (Brighteye Ventures, 2020). The growth is such that “EdTech started the decade with $500 m of Venture Capital investments in 2010 and finished 14 × higher at $7B in 2019”; while investment is exponentially growing since “85% of that was in the last 5 years and nearly 50% of the last decade’s funding occurred in the last 2 years”.^3^ Among investors and EdTech market intelligence organisations, the COVID-19 pandemic is recognised as a catalyst in an otherwise long-term trend of growth in online education. Consequently, the EdTech market “is expected to grow between 14.5% and 16.4% per annum to a total value of $368bn to $406bn in 2025” (IBIS Capital and Cairneagle Associates, 2020). In terms of venture capital investment, “EdTech attracted $4.5bn of VC through the first half of 2020, setting the sector up for a record setting full year”, while it is predicted that “$87bn will be invested in EdTech over the next 10 years, almost triple the prior decade”.^4^ These are impressive numbers, indicating an important dynamic in the HE sector and its digitalisation.

As of September 2020, there are 20 identified EdTech unicorns in the world, i.e. companies valued over $1bn.^5^ Out of those, nine had their last round of investment in 2020 (thus in the time after COVID-19 emergence), and 17 are spread between China and the USA. These two countries are dominating the digital economy more broadly and are indicative of the new power struggles in the global economic order (UNCTAD, 2019). Besides the impact of the COVID-19 pandemic speeding up the digitalisation of HE and the growth of EdTech (Williamson & Hogan, 2020), a vital driver for EdTech investment is a potential of turning traditional education into ‘Education-as-a-Service’ (Deepa & Sathiyaseelan, 2012). The opportunity recognised by investors and entrepreneurs lies in calculating the digital share in the global spending on education. Already in 2016, Ibis Capital, reported that education market was worth “over $5tn, 8 × the size of the software market and 3 × size of the media and entertainment industry, yet education is only 2% digitised”.^6^ In 2020, the digital share is 3.6% out of the $6.3 T spent on global education, while by 2025 the share is predicted to increase to 5.2%.^7^ Education is thus widely recognised as a lucrative opportunity, and coupled with COVID-19 urgency, renders EdTech and their digital models widely accepted in the sector.

Central business models in the digital economy are data-driven and enabled by digital platforms (Srnicek, 2017). These models only strengthened their presence since the global financial crisis of 2008 as tech giants and new platforms gained dominant levels of power and wealth (Sadowski, 2020a). Measured in market capitalisation, seven out of eight top companies in the world use platform-based business models (UNCTAD, 2019). Digital platforms are thus key and can be understood as “a distinct mode of socio-technical intermediary and business arrangement” (Langley & Leyshon, 2017, p.11). As intermediaries, they are connecting multiple actors and allowing them to interact. As business arrangements, they are creating multi-sided markets and coordinating network effects. Platforms are programmable and thus open to change by software developers for developing their own products, services, and marketplaces on a platform (Plantin, Lagoze, Edwards, & Sandvig, 2018), albeit to a more significant or lesser extent (Andreessen, 2007). As digital platforms are intermediaries and infrastructures at the same time, they have the advantage of recording and extracting all data on users’ actions and interactions. The “growth of digital platforms is directly linked to their capacity to collect and analyse digital data” (UNCTAD, 2019, p.xv).

Digital data is key to platforms and value in the digital economy, whose imperative is “extracting all data, from all sources, by any means” and as much as possible (Sadowski, 2020b, p. 9). This includes digital traces that we leave as part of our personal, social, and business activities. Digital platforms collect different kinds of personal data, spanning from content such as scholarly discussions in a virtual learning environment, to data on user behaviour such as users’ click-through of a platform, and metadata, such as data on users’ devices, location, and internet protocol address. Digital data is made valuable by extraction, enclosure, storage, aggregation, analysis, and transformation into intelligence (Savona, 2019). The current technical and legal arrangement allows turning the extracted personal data into private assets (Birch, Chiappetta, & Artyushina, 2020), to which I turn later in more detail.

#### A need for studying the diversity of digitalisation in and of higher education

Many digital platforms are present in the HE sector, supporting teaching and learning, research, management, and other processes. Some of them are developed by universities alone, while most are proprietary. Some are targeting institutions and enter the sector via new types of partnerships with universities (Thomas & Nedeva, 2018), while others are targeting individuals, i.e. students and teachers. Those platforms that are introduced into a university, plug-into its digital infrastructure (Williamson, 2018). This enables data flows between proprietary platforms and a university’s databases, which is as much a technical as it a legal issue. The complexity of digitalisation and marketisation of HE is thus high and works in different combinations (See Table 1).

Table 1 shows the diversity of actors who are part of digitalising HE, the variety of digitalisation forms in HE, and various monetisation models that are being established. The commonality among these forms is a shift from a business model of selling a commodity, which would involve a transfer of ownership rights for an exchange of a product or a service, to a business model based on a digital platform, which enables extracting value through ownership and control rights of digital data, and monetisation via rent. Monetary rent collected by digital platforms refers to various subscription fees, fees per click, time spent on a platform, and so forth.

The list is not exhaustive but rather presents instantiations of digitalising the HE sector dynamics. There might be new categories that are under development, such as universities developing digital assets and offering them to other universities for subscription fees or similar charges. This is a matter of much needed empirical focus that I spell out in this and next section of the article.

Table 1 uncovers the actors, who are part of the emerging platform ecology in HE and include universities, platform companies, and state agencies. Another layer of complexity is various relations between these different actors, either provider-user, business-to-business partnership, or other. While universities, students, and staff are digital platform users paying various fees in most of the identified instantiations, universities actively participate in profit-making in a new type of partnerships between public universities and platform companies (Ivancheva et al., 2020). OPMs support universities in developing and delivering courses online. The monetisation model is either that OPM companies share tuition revenue with universities over a certain period (usually a decade) for investing their resources in developing a course or charge a fee-for-service that universities pay (Perrotta, 2018). In case of MOOCs, the profits are made from charging fees for services other than access to content, i.e. fees for certification of attendance, certification of skills, corporate training, employee recruitment, and applicant screening (Taneja & Goel, 2014). There is an emergence of universities offering full degrees via MOOCs for a tuition fee or modules that users can stack-up into a degree.

Important to add to the list in Table 1 are investors in EdTech companies, who through financing, decide which services and products will be developed (Feher, 2018). They are also key actors to influence the specific business models of particular EdTech platforms and their profit strategies, by negotiating with entrepreneurs and making financial decisions (Muniesa et al., 2017). While there is emerging research on particular actors in EdTech, their motivations, and strategies, research on investors in EdTech is even scarcer (a recent study on lower levels of education was done by Regan & Khwaja, 2019). Second, the focus of attention in research thus far has not been the digital platform as the central socio-technical arrangement and the various business models behind it. Yet, these are key and consequential for HE. Crucial to analyse are, therefore, questions on who constructs these digital platforms, who owns them, and who controls them. These are as much technical as they are legal and financial questions (Sadowski, 2020a).

I propose a research focus on digitalisation of and in HE with the in-depth case-to-case analyses of different digitalisation forms, business and monetisation models, and partnerships and connection variations between public universities and platform companies. First, it needs to be recognised that there is a diversity of digital platforms with different monetisation and business models. Not all platforms are the same, and their owners differ in the way how they technically and legally control platforms and extracted data. Second, a careful analysis of these different monetisation models is needed, as identified in Table 1 and beyond. Finally, platform users pay monetary and data rent at the same time (Sadowski, 2020a). The monetisation models in Table 1 mainly refer to the monetary rent. However, we need theories that offer a better explanatory power for data rent than marketisation theories thus far used in HE research. I now move to data rent and theory of assetisation.

### Theoretical move: from commodification to assetization

Emerging studies on the intersection of digitalisation and marketisation of HE suggest these two processes are closely intertwined. However, they apply the theory of markets qua commodification, which might explain only parts of the digitalisation dynamics in HE. Instead, value in the digital economy lies in the extracted digital data, which is processed, monetised, and valorised in the form of assets and not commodities. Therefore, the difference between commodities and assets is paramount, including its theoretical underpinning, to which I turn next.

#### Commodities versus assets

Commodities are goods or services produced for and exchanged in the market. They are considered ‘real’ commodities if they are created for sale in the market (for example, a product like a car, or a service like a consultancy), and as ‘fictitious’ commodities if they are turned into a commodity form with the help of fiction (Polanyi, 1957) without being produced for the market, such as land, labour, money, and also knowledge (Jessop, 2007).

The market is typically understood as an institution for exchanging commodities, albeit there are many different markets in the plural, including markets for assets (Birch & Tyfield, 2013). While assets can indeed be bought and sold, the principal notion of their value realisation is rent, and not price. If one earns income from selling commodities by charging a price, the income from assets is mainly earned via collecting rents (see Table 2).

**Table 2:** Key differences between commodities and assets (Birch & Tyfield, 2013)

Commodity	Asset
An object (thing or service) produced for market exchange	A tangible or intangible resource that can be used to produce value and, at the same time, has value as property
One pays a price, which is a onetime payment for the exchange of ownership rights	One pays rent (such as a licence fee, a subscription fee, or a user fee), which is a continuous receipt of payments as a consequence of controlling access to an asset
Transfer of property rights with the exchange	Property and control rights stay with the asset owner
As value increases, demand decreases (as in classic supply–demand curves)	As value increases so does demand for it

Assets are legal constructs. Exercising ownership and control rights rests on the regulatory framework set by the state and international agreements (Dreyfuss & Frankel, 2015). However, through contract and property law, assets can entail the separation of the ‘thing’ and rights over it, which is not the case with commodities (Birch & Muniesa, 2020). While buying a commodity entails the transfer of ownership rights, assets have different modes of ownership and control. More specifically, owners of assets have exclusive legal rights to the use of the asset itself, as well as any copies derived from it. This is especially relevant with intellectual property (IP) rights like copyright, which is the central regime of governing digital platforms and data (Sadowski, 2020a).

The financial benefit of asset owners is perpetual into the future, as opposed to commodity owners who sell it once. Rights over reproduction thus enable constantly renewed resources (assets), which allow continuous income (rents) with negligible renewal or transactional costs (Birch, 2020). A commodity’s value is determined in a specific point of exchange via a price. An asset’s value is temporal and may change depending on a configuration of social, material, and discursive dimensions (Muniesa et al., 2017). Indeed, asset value is constructed in the light of expectations about future returns on investments. The critical determining factor is the struggle over whose expectations and whose imaginary of the future will be applied in asset valuation and development (Birch & Muniesa, 2020). Asset value and valuation are dynamic processes and constituted by constant power relations and negotiations between various social actors and their technologies, practices, tools, devices, rules, norms, and so on (Kornberger, Justesen, Koed Madsen, & Mouritsen, 2015). Devices such as business models become performative through these valuation processes (Doganova & Eyquem-Renault, 2009).

The extraction of economic rents is enabled by monopoly control (Birch, 2017; Mazzucato, 2018). As not all assets come in the same form, some are impossible to reproduce (such as land), while reproducing others may be prevented by law (such as the IP rights). In the case of HE examples above (Table 1), both apply. The assets in the form of digital platforms would be hard if not impossible, to reproduce because of the specificity of a particular infrastructure and its network effects (Srnicek, 2017). Moreover, reproduction of some of the platforms or their parts might be legally prevented if they are patented or issued copyright. Finally, assets have a different supply and demand logic than commodities due to their monopoly position, and the unique and specific characteristic of an asset. Rising prices of assets do not lead to competitors entering a market and driving the prices down (Birch & Tyfield, 2013). Also, increased demand does not necessarily drive the prices up. In the case of digital platforms, data use creates yet more data, and the network effects increase the value of these platforms (Srnicek, 2017). Moreover, while a commodity once used or consumed is gone, digital data is reproducible for almost zero marginal cost (Savona, 2019). Consequently, rentiership invites different behaviour and different motivation of asset owners in comparison to commodity producers.

#### Assetization and rentiership

The expanding digital economy is marked with the rise of rentiership, i.e. the appropriation of value through ownerships and control rights (Birch, 2020). Instead of entrepreneurial strategies based on commodity production, there is a focus on financial strategies of turning things into assets (Birch & Muniesa, 2020). In explaining what it takes to construct assets and rents, Birch proposes a theory of rentiership (Birch, 2020). He argues that rentiership involves a series of actions, knowledges, and transformations, which can make the realisation of value unfolding over several years or even decades. It includes a set of formal and technical, as well as more informal and normative processes.

Birch’s theory of rentiership comprises of three steps. The first, *‘thing-ification’ of knowledge*, starts from a position that knowledge is collectively produced and legitimated. For knowledge to be monetised and rents to be extracted, it has to be turned into a ‘thing’ and made alienable, such as securing a patent or issuing copyright. This process ensures that knowledge is separated from its function and can exist as property, which can be valued for future returns and rents. The second step refers to *turning things into assets*. I take Birch’s argument seriously that rents do not merely appear and exist as distortions in other political-economic processes. Instead, things are actively transformed into assets, which enable rent collection. Thus, we need to pay attention to the processes of how assets are made and how rents are constructed. In other words, after the thing is alienable and identifiable (step one), there is a question of what kind of asset form it gets turned into, and how. Finally, the last step, *capturing economic rents*, concerns how rentiership is enacted through different modes of ownership and control of diverse assets, including different follow-through rules that apply for various asset forms.

Assets come in different sizes, forms, and shapes. The kinds of assets relevant in the digitalisation of HE are intangible assets, and specifically, copyright and patents issued and secured by platform owners. These are protected by IP, giving owners exclusive rights, such as rights over access and exclusion, the right to copy software, create derivatives or modify versions, and distribute copies to the public by licence, sale, or otherwise. The software licence that platform users consent to in the form of ‘terms of use’, ‘terms and conditions’, and the like is the key technology to manage relations between platforms and their users (Birch et al., 2020; Sadowski, 2020a). There is a profound empirical gap in studying these processes in HE.

#### A need for studying data rents in higher education

In the above section on the empirical move, I suggested that HE scholars need to study the variety of digitalisation forms that are emerging in the HE sector; and the various monetisation models. In Table 1 I was referring to the monetary rents, such as subscription fees. However, platform users pay both monetary rent and data rent by leaving behind their digital traces. In this section, I suggest the future research should include a careful analysis of data rents in HE that we pay by leaving behind digital data coming from our engagement with and use of digital platforms.

Data rent refers to many different ways in which digital data extracted from digital platform users can be valorised other than simply turning it into money (Sadowski, 2019). For example, it can be made valuable by optimising systems, modelling probabilities and predictive analytics, profiling and targeting people via personalised advertising or political targeting, and managing people, organisations, or larger systems, and other data intelligence products and services (Sadowski, 2020b). As data is not rivalry in consumption, it can be used over and over again in different operations and combinations, and options are limitless (Savona, 2019). Indeed, there is an established marketplace for student data in which brokers collect and sell numerous combinations of data on students obtained from multiple sources (Russell, Reidenberg, Martin, & Norton, 2018).

To follow data rent models, instantiations of digitalisation identified in Table 1 should be studied in terms of who is the owner of extracted digital personal data, how the rights over data get to be exercised, and who profits from data aggregation and data analysis. It is safe to assume there are different ways of managing data rents. This could be because platform companies might have different motivations and business models, because they serve numerous users spanning from individuals to organisations, or because they make contracts with various institutional actors that lead to different arrangements.

In the case of targeting individual users directly, such as students and academics, terms of use apply, which are unilaterally issued by platform owners under the regime of copyright. If users want to access and use a platform, they must consent to the set terms. These terms have the status of a contract, which also include ownership and uses of extracted personal data (Lemley, 2006). Analysing terms of use issued by different EdTech platforms would contribute to understanding the varieties of constructing value from the extracted personal data. This is especially relevant because much of the EdTech investment is into companies offering products and services directly to users (Brighteye Ventures, 2020).

In the case of universities engaging with proprietary platforms as users, including platforms used by students and staff, universities negotiate and sign contracts with platform companies. Universities are obliged to make sure that all education and other legal requirements are met. At the moment, the legal frameworks more or less refer to data privacy in some parts of the world, and not to data value (Birch et al., 2020). In terms of value, different platforms likely offer different conditions to universities, depending on their business models. Some might allow access to extracted data from a particular institution to that institution, while others might not. Even if access is allowed, it is questionable how useful it would be for analysis, as value lies in large aggregation from many different institutions, and even merging of data points from various other sources. However, research about the university-platform arrangements is lacking. Future research must analyse these arrangements, how they organise ownership rights of extracted data, how they manage the use and monetisation of extracted data, and who pays what kind of rent for what service or product.

In the case of partnerships between universities and platform companies like OPMs and MOOCs to offer study programmes or their parts together, the most common model is to share the profits, which I elaborated above. However, it seems that the sharing of profit refers to monetary rent (Perrotta, 2018; Taneja & Goel, 2014). It is not known how the data rent is shared, if at all.

The political economy of digitalisation in and of HE thus far developing implies that it is most likely proprietary platforms who profit from the business models emergent in the digital economy by owning and controlling the rights over platforms and extracted data. Universities seem to participate in the lower end of value construction by keeping the rights over the digital content that their staff creates. Quite possibly, when universities deliver modules and courses in partnerships with the platform companies, they might be selling their services as commodities; while platform companies might benefit from assetising digital data and charging rent. Different platforms probably have different approaches with varying consequences for universities and individuals. A lot is at stake for the HE sector and its subjects, which makes the suggested research foci pertinent.

### Political move: from data privacy to data value

Digital technology has the potential to greatly benefit students, staff, universities, and HE sector at large. However, such positive impact is not automatic, and EdTech needs scrutiny and regulation. The way the digital economy has been developing thus far is marked with a particular form of rentiership, which is extracting value and is contributing to the digital divide, inequality, and uneven development globally (Birch et al., 2020; Sadowski, 2020b; UNCTAD, 2019). Not much is known about these trends specifically in HE. The emerging research found that partnerships between universities and OPMs and MOOCs in the UK and South Africa reinforce existing inequalities and stratification of HE systems (Perrotta, 2018; Swartz et al., 2019; Swinnerton et al., 2019). We indeed urgently need research on the political economy of EdTech, but we also need a public debate on what kind of HE digitalisation and EdTech we want.

The practice of governing digital platforms and extracted digital data, including in EdTech, with the pre-existing regime of IP rights, has superseded theory and policy (Savona, 2019). Some legal scholars criticise the current IP arrangements as outdated for the digital economy (for example, Kerber, 2016; Schneider, 2018; Wiebe, 2017). Moreover, the COVID-19 pandemic has initiated fast responses of education institutions and EdTech companies, coalitions amongst international and national actors, state-supported funding for schools and universities moving online, and wide adoption of emergency pedagogy with limited possibility for resistance (Williamson & Hogan, 2020). After the pandemic, the EdTech platforms will have been well embedded in universities’ and national HE digital infrastructures. On the one hand, this is a broader issue of the expansive digital economy faced by all economic and social sectors. HE will and should be part of the more general policy changes in the digital economy. On the other hand, the digitalisation of HE is fast evolving now. It has specific effects on the sector with possible profound long-term consequences (Williamson et al., 2020). That is why the policy-makers and HE actors, including universities, EdTech companies, and their investors, need to act fast to find legitimate and socially just solutions for the value capture and redistribution in HE.

Most of the debate on digital platforms and data so far has been around privacy, with the European Union’s General Data Protection Regulation (GDPR) recognised as the most advanced in the world (Birch et al., 2020). Universities and EdTech companies in the EU have to comply with it. The GDPR aims to give data subjects more power with individual rights, such as the right to erasure, the right to be informed of data use, and the right to data portability. Privacy is immensely important, especially in the light of surveillance (Marachi & Quill, 2020; Zuboff, 2019), and the digital economy’s imperative of controlling citizens and organisations by digital data and digital means (Sadowski, 2020b). However, the GDPR does not tackle the issues of value extraction and redistribution; as well as the impact of particular business models on the design of platforms found in HE.

When students and staff use platforms provided by universities for studying, teaching and learning, research, and other study or work-related purposes, it is the university who is responsible for contractual arrangements with platform companies. It is fair to assume that a university keeps ownership of its students’ and employees’ personal data; however, it might share non-identifiable personal data, which is not regulated by the GDPR. In other words, GDPR protects identifiable personal data and metadata, but much of it is non-identifiable and possibly shared with platform companies. It would be possible to share identifiable personal data too if contractual safeguards are in place (for example, see endnotes in Hewitt & Natzler, 2019). In countries where the GDPR does not apply, data sharing between universities and companies might be even more prevalent. Students and staff can become subject to nested data policies of different organisations protected by contracts, which are, however, likely to be subject to commercial sensitivity. Again, not much is known about these arrangements and needs to be empirically researched. Therefore, when studying data rent and its valorisation, HE scholars need to carefully analyse what kind personal data (all or non-identifiable; volunteered, observed or inferred; sensitive or non- sensitive) is shared, how, with whom, and for what purposes.

The political move of the proposed research programme should include changes in the governance of HE as a consequence of proprietary platform expansion. Arrangements between platforms and users, as well as between platforms and universities, are subject to contract law. As users have little choice but to consent to platforms’ terms of use should they wish to access the platforms, authors critique this regime as disempowering users (Birch et al., 2020). The governance of education activities mediated by platforms seems to be shifting from public education law and public scrutiny, to contract law and commercial sensitivity for much of the digitalisation processes in HE.

Finally, we need research on the fast emerging practices in HE to contribute to the political debate on how to conceptualise, govern, and regulate value construction and redistribution in the digital economy more broadly, and in HE specifically. In terms of value construction and redistribution, there have already been attempts to organise customer to business data markets, in which users would be financially compensated for the data that was extracted. These have been unsuccessful since individual data is deemed not valuable, and it becomes such only when de-identified, aggregated, and analysed (Beauvisage & Mellet, 2020). For the future policies, authors offer different proposals on how to conceptualise personal data, namely as labour, capital, or IP, and each would have a different value redistribution and governance arrangement (for a summary, see Savona, 2019). There are also more substantive proposals on making extracted data freely available for the greater social benefit (Morozov, 2019; Sadowski, 2020b).

These are the policy debates yet to be held in future. Some sectors in the EU have already made specific arrangements for data sharing beyond the GDPR’s right to data portability. However, an overall framework would be needed (Graef, Husovec, & van den Boom, 2019). In 2020, the European Union is developing a new Digital Services Act; however, it is not known if or how it might include questions of data value and data sharing across different economic and social sectors (Streel & Husovec, 2020). Looking beyond the European Union, global policy coordination is necessary to address concerns of the digital gap and equality, and unevenness in value capture from digital data (UNCTAD, 2019).

## Conclusion

This article focuses on the political economy of EdTech and extracted digital data in the HE sector. While there is much research on technology and teaching and learning processes, there is less research on the issues around privatisation, monetisation, and new forms of value related to HE digitalisation, which this article addresses.

I first argued that digitalisation of HE needs to be understood as part of a broader expansion of the digital economy. This is crucial as the digital economy brings new forms of value construction based on data extraction, analysis, and intelligence, and governed by IP rights. The key business models are enabled by digital platforms, which are a specific socio-technical arrangement, and function as intermediaries and infrastructure. Platform owners make most profits not by selling commoditise, but by charging rent. I then argued that there are different forms and types of HE digitalisation, most of which include proprietary platforms. While universities may operate under the markets qua commodification, platforms seem to operate under markets qua assetisation.

Second, I argued that a theory of rentiership (Birch, 2020) and the processes of assetisation (Birch & Muniesa, 2020) provide better explanatory power to analyse the current HE digitalisation, than the marketisation theory used by most of the HE literature thus far. I presented the differences between commodities and assets. Digital platforms and extracted data are constructed as assets and besides monetary rent, also collect data rent. I suggested a new research agenda involving a careful, in-depth, case-to-case analyses of different assetisation processes, the kinds of assets that are created, and the kinds of monetary and data rents that are collected. In particular, I explained the importance of analysis of extracted data ownership, and contracts between platform owners and universities, as well as between platform owners and individual students and staff.

Finally, I argued that while data privacy is crucial, it should not be our only concern. From a political–economic perspective, the equal focus of interest should be data value. While practice superseded theory, there are emerging debates among some policy actors around digital data value redistribution and potential regulation. It will be crucial that HE stakeholders can participate in these debates aided by future research suggested in this article.

## Footnotes

1 EdTech market is worth $187bn with about 15% growth rate as estimated by IBIS Capital, a specialist investment and corporate finance advisory group focused on media, education, and healthcare sectors: https://www.ibiscap.com/index.php/insights-research/ (Accessed 18 September 2020).

2 The estimations are provided by Credit Suisse, an investment bank, wealth manager, and financial services provider: https://www.credit-suisse.com/ch/en/articles/asset-management/covid-19-is-a-powerful-catalyst-for-edtech-202007.html (Accessed 18 September 2020).

3 These figures are provided by Holon IQ, an international EdTech market intelligence company: https://www.holoniq.com/notes/87bn-of-global-edtech-funding-predicted-to-2030/ (Accessed 18 September 2020).

4 Estimates are provided by Holon IQ in its predictions on the impact of the COVID-19 pandemic on venture capital investment in EdTech: https://www.holoniq.com/notes/4.5b-global-edtech-venture-capital-for-q1-2020/ (Accessed 18 September 2020).

5 The list of EdTech unicorn companies is provided by Holon IQ: https://www.holoniq.com/edtech-unicorns/ (Accessed 18 September 2020).

6 As reported by Finance Digest: https://www.financedigest.com/global-report-predicts-edtech-spend-to-reach-252bn-by-2020.html (Accessed 18 September 2020).

7 In August 2020, Holon IQ updated their predictions affected by the COVID-19 pandemic: https://www.holoniq.com/notes/global-education-technology-market-to-reach-404b-by-2025/ (Accessed 18 September 2020).
